# Comparison of AH26 Physicochemical Properties with Two AH26/Antibiotic Combinations

**Published:** 2010-02-20

**Authors:** Hasan Razmi, Shaghayegh Parvizi, Azam Khorshidian

**Affiliations:** 1. Department of Endodontics, Dental School, Dental Research Center, Tehran, University of Medical Sciences, Tehran, Iran.; 2. Dentist, Dental Research Center, Tehran, University of Medical Sciences, Tehran, Iran.

**Keywords:** AH26, Amoxicillin, Doxycycline, Physicochemical Phenomena, Root Canal Filling Materials, Solubility, Viscosity

## Abstract

**INTRODUCTION:**

The purpose of this study was to compare the setting time and post-setting solubility, flow, film thickness and dimensional changes of AH26 root canal sealer with AH26-Antibiotic combination.

**MATERIALS AND METHODS:**

This study was performed according to British standard BS 6876 (2001) which tests the physicochemical properties of endodontic sealers. Three samples of each of tested materials including AH26 alone, AH26/amoxicillin and AH26/doxycycline were used to test each of the properties. They were prepared according to ISO protocols.

**RESULTS:**

The setting time of studied materials was 46 hours for AH26, 29 hours for AH26/amoxicillin, 49 hours for AH26/doxycycline. Flow test results were as follows, for AH 26, 15.6 mm; AH26/amoxicillin, 14.9 mm; AH26/doxycycline, 14.2 mm. Film thickness was 0.024 mm in AH26, 0.0283 mm in AH26/amoxicillin, 0.0276 mm AH26/doxycycline. The solubility of AH26 was 0.0076%, AH26/amoxicillin, 0.0113%, and for AH26/doxycycline, 0.013 %. Dimensional changes following setting was 0.07 mm, 2.6 mm, and 1.1 mm for AH 26, AH26/amoxicillin, and AH26/doxycycline, respectively.

**CONCLUSION:**

The physico-mechanical properties of AH26 antibiotic combinations were superior compared with AH26, with the exception of flow. Also, AH26/amoxicillin had a lower setting time than AH26. However, all values were within an acceptable range which conformed to ISO.

## INTRODUCTION

Biomechanical debridement of the root canal system is one of the leading tasks in endodontic treatments. Suitable instrumentation, irrigation and intracanal medication significantly reduced microorganisms inside the infected root canal system. Some studies have also documented bacterial presence in the dentinal and cementum tubules [1][2].

Although there are several reasons for endodontic treatment failure, studies have discovered that the presence of bacteria is the main cause for failure [3]. Using antibacterial drugs in root canal obturation, may allow the spread of the medicinal agents throughout the dentinal surface, root canal irregularities, apical foramen and periapical tissues; thereby decreasing bacterial presence and encouraging healing [4].

In chronic alveolar infection following tooth pulp necrosis and periradicular tissue degeneration, antibiotics cannot reach the entire infected area due to insufficient blood supply. Intra canal antibiotics may be more efficient and possibly superior to systemic administration as this will avoid side effects and permit higher concentration of antibiotic to reach the infected area [5].

In the case of pulp necrosis and apical periodontitis, choosing suitable sealer with high antibacterial activity can decrease or prevent growth of remnant microorganisms Enterococcus (E) faecalis is an anaerobic gram[6].

Positive bacteria often found in endodontic failure/chronic periapical lesions [7][8][9].

Slutzy-Goldberg investigated the antibacterial effect of sealers and showed that AH plus and RoekoSeal were ineffective on E. faecalis [10].

Miyagak showed that AH Plus and N-Rickert have antimicrobial effects against C. albicans, S. aureus and E. coli; however, E. faecalis was resistant against all tested sealers [11].

There are different studies analyzing the sensitivity of E. faecalis to various antibiotics such as amoxicillin, vancomycin, erythromycin, benzyl-penicillin and doxycycline [12][13][14].

The different physical and clinical properties may be examined by laboratory tests, this includes flow of sealer, solubility, working and setting time. Sealers need sufficient working time for appropriate clinical application in root canals. Flow measurements evaluate the ability of sealer to fill accessory canals, main canals and the space between gutta-percha points. In addition, as the unset sealer may cause harmful tissue reactions, the shorter setting time of sealers is favorable. Film thickness and solubility are also two major factors which influence sealing ability [15][16].

AH26 sealer is the most commonly used resin sealer introduced by Schroder as a root canal filling material. AH26 is able to effectively adhere to the dentinal walls, it has favorable flow and working time, low solubility and shrinkage and suitable compatibility with human tissues. Although the physical and mechanical properties of the sealers have been investigated [17][18], limited studies have focused on sealer and antibiotic combinations. One such study analyzed antibacterial effect of sealer-antibiotic combination with agar diffusion test [19].

In the present study, amoxicillin and doxycycline were used, due to the widespread use of amoxicillin in endodontics [20] and the prolonged antibacterial activity (bonding to the dentine) of doxycycline [21]. The aim of this study was to investigate changes in the physical properties of AH26 including solubility, setting time, working time, dimensional change after setting, flow and film thickness.

## MATERIALS AND METHODS

The pilot test determined the lowest effective concentration which the two sealer-antibiotic combinations can be used to inhibit bacterial growth. One percent of sealer weight was determined to be sufficient antibiotic material [22]. According to BS EN ISO 6876: 2001, the physical and mechanical properties of the sealers including setting time, solubility, flow, film thickness and dimensional changes following setting were investigated in the three group: 1-AH26 (Dentsply, DeTrey, Konstanz, Germany) 2-AH26 and Amoxicillin (Dentsply, DeTrey, Konstanz, Germany) combination 3-AH26 and doxycycline (Iran Daru, Iran) combination, [23].

All five tests were carried out for the three samples of each group as follows:

***1- Flow test:***

Approximately 0.05±0.005 mL of sealers was prepared according to the manufacturer's instructions. The sealer was placed on the center of glass plate (40×40×5 mm in sizes) by using a graduated syringe. After 180±5 sec of mixing, the second glass plate (100 g) was centrally placed over the sealer with an additional weight to achieve a total of 120±2 gr on application. After 10 min, the sealer was removed and the minimum and maximum diameters of compressed disk were measured; if these two numbers were within 1 mm (i.e. valid result), the average value was recorded. If the sealer disc was not circular or the max/min was not within 1 mm, the test was repeated. Three valid tests were required to attain an average.

***2- Setting time:***

Three stainless steel ring shape moulds with an internal diameter of 10 mm (cavity diameter) and 2 mm height were prepared. Sealer was prepared according to the manufacturer's instructions. Stainless steel moulds were placed on the glass plate; subsequently moulds were filled with sealer to leveled 1mm thicknesses. Approximately 120±10 sec after mixing, these samples were placed on a metal block (with 20×10×8 mm dimension) in a cabinet with 95% humidity and 37±1˚C temperature. When the setting time approached, an indenter instrument (100±0.5 g) which had (2±0.1 mm diameter) a flat cylindrical tip, was dropped vertically over a distance of at least 5 mm on to the horizontal surface of the sealer. The tip is cleaned and the operation is repeated until no indentation can be seen. The time of “no indent” is recorded from the end of mixing. The average value is known as the setting time.

***3- Film thickness:***

Two glass plates with a minimum uniform thickness of 5 mm and 200±10 mm2 contact surface were placed on each other and the combined thicknesses of two plates was measured with micrometer to an accuracy of 1 µm. Sealers were prepared according to the manufacturer's instruction and 0.5 mL of sealer was placed on the center of one plate. The other plate was placed on the sealer. After 180±10 sec post mixing, a vertical load of 150 N was applied on the plate so that the sealer can fill inter-plate area completely. Ten minutes after mixing, total thickness of the two plates and the sealer film was measured with a micrometer. The difference between two measurements showed the thickness of sealer. This test was performed 3 times and an average was recorded.

***4- Dimensional changes following setting:***

Three split cylindrical moulds made of stainless steel or other compatible materials with 6-mm diameter and 12-mm height were prepared. The moulds were placed on a glass plate (75×25×1 mm3 equivalent to microscope slide) which was wrapped with polyethylene sheet. The mould was filled with sealer to slight excess. Another glass plate was pressed on the mould so that it faced with polyethylene sheet. This collection was joined using a C-shape clamp. Five minutes after mixing the sealer, the joint set was transferred to an incubator (humidity= 90%-100% and temperature=37±1˚C). Samples were placed in the incubator for 3x the setting time. Then the ends of the samples were smoothed by back and forth motions across 600 grit wet sand paper, to achieve a flat surface. Samples were removed from the moulds and the distance between two flat ends was measured to an accuracy of 10 µm and stored in distilled water at 37±1˚C. After 30 days, the measurements were repeated and dimensional change was determined as the percentage change in length.

***5- Solubility:***

One split-ring mould with an internal diameter of 20±1 mm and a height of 1.5±0.1 mm was prepared and placed on the glass plate with larger dimensions than the mould; the mould was then filled with sealer. Another glass plate lined with plastic sheets (polyethylene) was pressed on the sealer, and carefully removed in order to remain a flat uniform surface. The filled mould was placed in an incubator with 37±1˚C temperature and 95% humidity, 50% longer than the recorded setting time. Sealers were removed from the mould and the weight of sealer was measured to the nearest 0.001 g. Two of these samples are placed in shallow dish with diameter of 90 mm and minimum volume of 70 mL. Samples were not touched by hand. Approximately 50±1 mL of water was added and covered the dish. The dish was kept in a incubator for 24 hrs; the samples were then removed and washed with 2 to 3 mL of fresh water. The presence of particles was evidence of disintegration and therefore was not accepted. Samples were separated to the final mass. The water was evaporated without boiling by drying the dish in an oven (110±2˚C), it was then cooled to room temperature in a desiccator (phosphorus pentoxide). The amount of sealer which had dissolved equaled the difference between the first mass and final mass of dish. Tests were repeated twice and the average was recorded to the nearest 0.1%. Solubility was calculated from this formula: (W0 - Wf):W0; W0 was the initial weight and Wf was the final weight of the sample.

## RESULTS

Table 1 shows the flow and film thickness results. Table 2 presents the setting time of sealers. Dimensional changes and solubility after setting of sealers are exhibited in Table 3.

**Table 1:** The means of flow (F) and film thickness of studied sealers

**Sealer **	**Mean film thickness (mm) **	**Mean flow (mm) **
**AH26**	024	15.6
**AH26/amoxicillin**	0283	14.9
**AH26/doxycycline **	0276	14.2

**Table 2:** Setting time (S.T) of studied sealers

**Sealer**	**Setting time(hour)**
**AH26**	46
**AH26/amoxicillin**	29
**AH26/doxycycline **	49

**Table 3:** Solubility and dimensional changes after setting of sealers

**Sealer**	**Solubility** **(%)**	**Dimensional** **changes (%)**
**AH26**	0.0076	0.7
**AH26/amoxicillin**	0.0113	2.6
**AH26/doxycycline **	0.013	1.1

In this study, the film thicknesses of the sealers were measured as 0.024, 0.0283, and 0.0276 mm for AH26, AH26/amoxicillin and AH26/doxycycline, respectively.

The average flow of AH26, AH26 with amoxicillin and AH26 with doxycycline were 15, 14.9 and 14.2 mm, respectively.

Setting times of AH26, AH26 with amoxicillin and AH26 with doxycycline in this study were 46, 29 and 49 hours, respectively.

In this study, the film thicknesses of the sealers were measured as 0.024, 0.0283, and 0.0276 mm for AH26, AH26/amoxicillin and AH26/doxycycline, respectively. Solubility percentages of AH26, AH26/amoxicillin and AH26/doxycycline were 0.0076%, 0.0113% and 0.013%, respectively.

## DISCUSSION

Intracanal medicament can significantly assist biomechanical instrumentation and root canal irrigation efficacy in reducing micro-organisms in infected canals [1][2]. Root canal sealers with antibacterial properties can prevent and reduce bacteria growth. Suitable characteristics of AH26 sealer, i.e. low solubility and shrinkage, ideal biocompatibility, sealing ability, acceptable working time and penetration into dentinal tubules has made this sealer popular [24]. The greater the penetrating ability of sealer into dentinal tubules, the greater capability to trap bacteria and prevent their growth [6]. Sealers’ flow and penetrative ability between gutta-percha cones, and into the accessory canals is very important [25]. This study showed that the flow of AH26, AH26 with amoxicillin and AH26 with doxycycline is in accordance with BS EN ISO 6876 (2001) standards and their average flow were 15, 14.9 and 14.2 mm, respectively.

There is no definite standard for the setting time of root canal sealers; the clinician will however, required sufficient working time for material placement [25]. Excessive time is not favorable as this may dry the canal [26]. In this study, all studied sealers had setting times longer than those claimed (9-10 hours) by the manufacturer (Dentsply, DeTrey, Konstanz, Switzerland). Setting times of AH26, AH26 with amoxicillin and AH26 with doxycycline were 46, 29 and 49 hours, respectively. Takechi et al. illustrated that adding Flomoxef sodium antibiotic to the calcium phosphate cement can lead to a longer setting time [27]. Mcmichen et al. compared film thickness of some sealers and showed that the highest film thickness was 0.44 mm for AH-plus and 0.12, 0.18 and 0.11 mm for other sealers [25]. Lacey et al. showed that there was no significant difference in the viscosity of endodontic sealers, by increasing the temperature from 25˚C to 37˚C [28].

In this study, the film thicknesses of the sealers were measured as 0.024, 0.0283, and 0.0276 mm for AH26, AH26/amoxicillin and AH26/doxycycline, respectively.

A thin film thickness is superior to a thick film sealer due to its better penetrability and thus provides a better seal. The film thickness values of both sealers obtained in this study conformed to British standard BS 876 (2001).

Sealers contact with liquids such as water can cause dimensional changes following setting. Dimensional changes of AH26, AH26/amoxicillin and AH26/doxycycline were 0.7%, 2.6% and 1.1%, respectively. Versiani et al’s values were similar, i.e. 1.3%, and 8.1% for AH-Plus and Epiphany sealers, respectively [29].

The quantity of a particular substance that can dissolve in a particular solvent (yielding a saturated solution) is called its solubility under specified temperature and pressure. Mcmichen showed that AH26 sealer had the lowest solu-bility compared to Apexit, Endion and Tubli-Seal EWT; Apexit had the highest solubility [25].

Schafer discovered that AH26 and AH-Plus sealers had less solubility in water and artificial saliva compared to other sealers i.e. Apexit, RoekoSeal, Sealapex, Diaket and Ketac Endo [18]. In our study, solubility of AH26, AH26/amoxicillin and AH26/doxycycline were 0.0076%, 0.0113% and 0.013%, respectively. This demonstrates that the addition of antibiotics significantly increased solubility; which is an unfavorable response.

## CONCLUSION

According to this study, enhancing sealers with antibiotics led to a decrease in flow of the sealer, changes in setting time of AH26 i.e. decrease in setting time with amoxicillin and increase with doxycycline, increase in film thickness, increase in the dimensional changes following setting, and increase in the solubility of the sealers. However, all changes were still within the BS EN ISO 6876 (2001) standards.
